# Importance of Meteorological Parameters and Airborne Conidia to Predict Risk of *Alternaria* on a Potato Crop Ambient Using Machine Learning Algorithms

**DOI:** 10.3390/s22187063

**Published:** 2022-09-18

**Authors:** Laura Meno, Olga Escuredo, Isaac Kwesi Abuley, María Carmen Seijo

**Affiliations:** 1 Department of Vegetal Biology and Soil Sciences, Faculty of Sciences, University of Vigo, As Lagoas, 32004 Ourense, Spain; 2 Department of Agroecology, Flakkebjerg Research Center, Aarhus University, Forsøgsvej 1, 4200 Slagelse, Denmark

**Keywords:** aerobiology, *Solanum tuberosum*, early blight, *Alternaria* spp., weather factors, machine learning, k-nearest neighbor, random forest, decision trees

## Abstract

Secondary infections of early blight during potato crop season are conditioned by aerial inoculum. However, although aerobiological studies have focused on understanding the key factors that influence the spore concentration in the air, less work has been carried out to predict when critical concentrations of conidia occur. Therefore, the goals of this study were to understand the key weather variables that affect the hourly and daily conidia dispersal of *Alternaria solani* and *A. alternata* in a potato field, and to use these weather factors in different machine learning (ML) algorithms to predict the daily conidia levels. This study showed that conidia per hour in a day is influenced by the weather conditions that characterize the hour, but not the hour of the day. Specifically, the relative humidity and solar radiation were the most relevant weather parameters influencing the conidia concentration in the air and both in a linear model explained 98% of the variation of this concentration per hour. Moreover, the dew point temperature three days before was the weather variable with the strongest effect on conidia per day. An improved prediction of *Alternaria* conidia level was achieved via ML algorithms when the conidia of previous days is considered in the analysis. Among the ML algorithms applied, the CART model with an accuracy of 86% were the best to predict daily conidia level.

## 1. Introduction

Early blight caused by *Alternaria solani* (Soraeur) and *A. alternata* (Fr.) Keissl is an important disease for potatoes production globally. Driven by the rapidly changing climate, the disease’s intensity has increased in several areas recently. Consequently, massive yield losses are happening globally [1,2]. Generally, growers spray chemical fungicides routinely to achieve effective control of the disease. To regulate the number of sprayings and minimize problems, such as contamination of the environment and fungicide resistance, there is the need to regulate the application of fungicides via decision support systems (DSS) [1,2,3,4,5,6].

The resident inoculum in the field from previous cropping seasons plays a vital role in the onset of early blight [3,7], but the subsequent development of the epidemic in the field is conditioned by aerial inoculum and weather conditions. Traditional DSS models do not explicitly predict conidia concentration in the air or the presence of resident inoculum in the field from previous cropping seasons or neighbor potato crops. However, aerobiological studies, which study aerial biological elements and the factors influencing them, can be useful to inform us about *A. solani* and *A. alternata* conidia levels in the air and, therefore, for understanding the airborne conidia dynamics. Previous aerobiological studies on *Alternaria* spp. have focused on seasonal conidia variation in urban environments [8] or rural environments [9,10], whereas other studies have been carried out on the intra-diurnal pattern of *Alternaria* conidia [6,9,10,11,12,13,14]. Studies on intra-diurnal variation are becoming more interesting, and some papers have shown that conidia concentrations vary considerably between 3 and 30% over a 24-h period [11,13]. Intra-diurnal variations of airborne fungal spores depend, among other factors, on weather conditions that affect the sporulation, dispersal, and deposition of spores [15]. Changes in temperature during a day can favor thermal turbulence, which simultaneously dilutes spore concentrations at ground level and causes the release of more spores by mechanical disturbance [15]. Active mechanisms of spore discharge are also related to rapid changes between wet and dry conditions. Dry spore-discharging fungi release spores by the flow of air or by hygroscopic twisting movement, which occurs upon drying. Such spores are mostly emitted when dry, warm, and windy conditions prevail [16].

Aerobiological studies have focused on understanding the key factors that influence the spore or pollen concentration in the air through correlation analysis [2,10,17,18,19,20,21], but less work has been carried out to predict when critical concentrations of conidia occur [22]. Indeed, understanding the factors associated with conidia dispersal is relevant, but predicting when a significant number of conidia occur will be more useful for practical disease control (for pathogenic species) in agricultural systems. Some studies have focused on trying to predict the *Alternaria* concentration in the air by using linear regressions or ARIMA [4,17,21]. However, the dispersal of conidia in the field is affected by a multitude of weather factors, which complicates its prediction with simple data analyses.

Machine learning (ML) has emerged along with other technologies (big data and high-performance computing) to create new opportunities for big data analysis obtained in intensive processes in agricultural operating environments and others. Currently, ML is applied in many scientific fields, including meteorology [23,24,25], food safety [26], climatology [27,28], and aerobiology [21,29,30]. One important field in which machine learning and artificial intelligence can be used is in plant pathology, to detect plant diseases and pests with digital images [31,32].

The use of ML algorithms (e.g., decision trees, random forest, k-nearest neighbor) in field studies provides us with the tools needed to achieve such predictions. These algorithms can identify patterns in the occurrence of an event (e.g., conidia) and the factors that affect the event and, thus, can be used for predictive modeling. In this sense, ML can be useful for predicting the *Alternaria* conidia dispersal in the field and the most influencing weather variables on conidia release. These ML algorithms, such as classification and regression trees (CART), have been used for understanding the factors affecting conidia [14,20,21], but not for predicting them. The prediction of conidia concentrations in the atmosphere is key to minimizing the use of fungicides and practical disease control and, thus, this is achievable via ML algorithms. The presence of inoculum in the air could be integrated into disease management or DSS in two ways. Firstly, intra-diurnal variation of conidia according to weather parameters can be studied and used to decide on the best time to apply fungicide relative to the critical period in the day in which conidia concentration is highest. Secondly, the daily conidia level could be predicted to determine when critical levels of conidia are likely to occur according to weather parameters and, thus, fungicide must be applied when the threshold is reached [4,17].

A Limia (NW Spain) is one of the most important potato producing areas in the country. The majority of the agricultural land is cultivated using traditional methods highly dependent on the use of pesticides, but the deterioration of rural areas and the incorporation of a young generation of farmers looking for a greener agriculture has promoted significant changes in crop management. There is particular interest concerning innovative technologies for plant breeding and the introduction of remote sensing for different purposes. In the last growing seasons, *Alternaria* epidemics are becoming more frequent and aggressive, requiring several applications of specific fungicides for its control [2]. Farmers demand research and innovation in the management of this disease. Despite several studies were carried out in relation to the progression of early blight in the area [2,4,10,17] and to adjust decision support models proposed for other geographical areas, there are no studies using ML algorithms to predict daily *Alternaria* conidia. The results obtained can be easily extrapolated to other geographical areas.

Therefore, the objectives of the present study were as follows: (1) to understand the key weather variables that affect the hourly conidia dispersal of *A. solani* and *A. alternata* in a potato field; (2) to study the daily weather variables that influence daily conidia concentration in the air; (3) to use these factors in different ML algorithms to predict the daily conidia levels.

## 2. Materials and Methods

### 2.1. General Aspects of the Experimental Field

The experimental field was located in A Limia (Galicia, Northwest Spain) a geographical area where potatoes and wheat in annual rotation are the main agricultural crops. The study was done for five growing seasons (2017–2021). Each year, a 4-hectare field was planted with the potato cultivar Agria. This potato cultivar is susceptible to early blight. The planting dates and dates of the important phenological stages for each year are shown in Table S1. Weather data and aerobiological data were recorded from crop emergence to crop senescence for each potato crop season.

### 2.2. Weather Data

Weather data were registered during the entire crop cycle of each year using a portable weather station (i-METOS) placed at 1.5 m height in the middle of the experimental field. Data of temperature (Temp, °C), dew point temperature (DewTemp, °C), relative humidity (RH, %), wind speed (Wind, m/s), rainfall (Rain, mm), solar radiation (Rad, W/m^2^), and leaf wetness (LW, h) were recorded hourly. The weather station also provided daily data for the variables mentioned before.

### 2.3. Aerobiological Sampling

A Lanzoni aerobiological sampler (Lanzoni S.r.l., Bologna, Italy) with a 7-day recorder spore-trap was placed 1.5 m away from the weather station. This sampler is a unit with an in-built vacuum pump, which is designed to sample airborne particles (fungal spores in this study). The sampler contains a clockwork-driven drum with a Melinex tape covered by an adhesive substance where particles impact. This device allows us to obtain hourly data concerning conidia. The methodology used for the aerobiological count of conidia was described by Galán et al. [33]. The conidia of *A. solani* and *A. alternata* were counted, and the concentration was expressed as conidia/m^3^.

The release of conidia from the conidiophore (expressed as spore release [SR]) was calculated with hourly RH data, and the escape of conidia from the crop canopy into the atmosphere (named as “Escape”) was calculated with the hourly wind speed data according to the formula proposed by Skelsey et al. [15].

### 2.4. Phenological Study

The stages of crop development were monitored weekly from plant emergence until crop senescence. For this, the potato crop development was divided into three main phenological stages based on the BBCH scale of the potato crop, as follows [34]:

1. Vegetative stage—the period from 50% crop emergence until the beginning of flowering (BBCH 09–61);
2. Reproductive stage (flowering)—the period from which at least 50% of plants were flowering until when the flowers begin to fall (BBCH 61–69);
3. Senescence stage—when 50% of the plants begin to yellow/die until when 50% of plants were completely dead (BBCH 95–99).

### 2.5. Data Analyses

Data preparation and statistical analyses were performed with the R language and environment for statistical computing version 4.1.3 [35] using R software (R Language and Environment for Statistical Computing [version 4.2.1]). The following two types of data were used in the statistical analysis: hourly and daily.

#### 2.5.1. Hourly Analysis

Graphical model to ascertain the correlation structure between the hourly weather data and conidia was used as described previously [36,37]. The graphical model was carried out with dataset by minimizing the Bayesian information criterion (BIC) with the grapHD R package (version 0.2.0) [38] implemented in R software. The theory of this method is based on the conditional independence between a set of random variables provided in the dataset. The covariance structure of a range of variables is encoded in a set of vertices represented by points (variables) and a set of edges or lines connecting the vertices. The pair of variables for which the conditional correlation given the other variables, is significantly different from zero are connected by an edge (line). In other words, a pair of vertices connected by a line are significantly correlated and vice versa. After the graphical model, a correlation analysis to determine the specific correlation (negative or positive) between the weather variables and conidia per hour with the ggcorplot R package version 0.1.3 [39] was carried out. Spearman rank correlations between hourly conidia concentration and weather parameters were considered.

Finally, the weather and conidia data for the five crop seasons were pooled, and their mean values were computed. This was performed to analyze the overall effect of hourly weather values, as well as the hour of the day, on the intra-diurnal conidia concentration. To achieve this, the correlation structure of the variables was analyzed through graphical modeling. Subsequently, a linear regression with the variables that were found to be significantly correlated with the conidia was calculated. The significance level was set at α = 0.05.

#### 2.5.2. Daily Analysis

For analysis of the daily data set, the conidia and weather data of the five crops seasons were used. First, a graphical model to determine the correlation structure between the variables was carried out. This was followed by a specific analysis of the Spearman correlation between the daily variables of the current day and the data of four previous days (−1, −2, −3, −4). The daily data set was used to predict conidia levels. Conidia level was defined as the concentration of conidia that can cause considerable infection in the field, considering a threshold of 10 conidia/m^3^. Then, the days with conidia concentration lower than this threshold were classified as unmeaningful (UM), whereas the days that exceeded this conidia concentration were classified as meaningful (M). This threshold was chosen based on experiences in the field and has been also used previously to forecast *Alternaria* concentrations [22]. Next, the data set was split into two groups (one which includes conidia and one which did not). Different ML algorithms to develop a predictive model for conidia level separately were used, namely decision tree, k-nearest neighbor (KNN), and random forest (RF).

Decision trees represent relationships between predictors and potential outcomes using a tree-like structure [40]. The tree starts with a single node (i.e., root), followed by progressively smaller partitions as it grows. Each time the tree splits, the decision is made as to how to partition the data based on the values of the predictor. The split points are called decision nodes, and the outcomes are called branches. Further partitioning of the data produces new decision nodes, which in turn produce additional decision nodes until the decision tree ends. Nodes at the end or terminal of a tree are called leaf nodes. Leaf nodes represent the predicted outcome based on the set of decisions made from the root node, through the decision nodes to the leaf node [40]. For our study, we used the following two decision tree models: (a) “rpart” (recursive partitioning regression tree), which is also called a classification and regression tree (CART), and (b) the “C5.0” method. One of the distinguishing features between the two decision tree algorithms is how they measure impurity during the learning process. While the C5.0 algorithm uses entropy (i.e., a measure of the randomness in a partition), CART uses Gini (i.e., a measure of the frequency that a particular data point in a partition would be incorrectly labeled if it was assigned a random label based on a distribution in the data partition).

The KNN method belongs to a family of algorithms known as lazy learners since they do not build a model or learn anything. To assign labels to unlabeled data, they simply refer to the training data during the prediction phase. In KNN, the distance between the test data and the new instance is computed. The closest K data points in the training dataset are found based on certain distance functions. Here, K is the number of nearest data points (neighbors) [41].

Random forest algorithms (also known as decision tree ensemble algorithms) combine the results of multiple independent decision trees to make predictions about new data sets [42]. Each tree in the forest assigns the most probable class label to each input. Random forests are generally robust and stable compared to single trees built by decision trees, such as classification and regression trees (CART). However, a major shortcoming of this algorithm is that is not easily interpretable compared to CART.

##### Implementing the ML Algorithms

The data sets (without or with conidia) were split into 80% (411 values/rows) training and 20% (102 values/rows) test data sets. All the algorithms were implemented with the “train” function in the caret R package version 6.0-92 [40]. The method option in the “train” function was set to “rpart”, “C5.0”, “knn”, and “rf” for implementing the CART, C5.0, KNN, and RF algorithms, respectively. For all algorithms, we used 10-fold cross-validation (CV) to optimize the models. The decision tree using the CART model was optimized (i.e., pruned) by selecting an optimal complexity parameter (cp) via CV. For the C5.0, we evaluated both the tree and rule-based models with or without winnowing (i.e., a process of removing uninformative predictors), and the best model was selected. For the KNN, the optimal number of neighbors (i.e., K) was selected via evaluation of a range of possible Ks via CV, and the best one was selected. The hyperparameter (i.e., node size) in the RF algorithm was also optimized by comparing the accuracy of models from different node sizes (1 to 10). The node size that resulted in the highest accuracy was selected for building the RF model.

The following metrics were used to evaluate the models: accuracy (i.e., the percentage of correct predictions by the model), and kappa statistics/accuracy (i.e., as an adjustment to predictive accuracy by accounting for the possibility of a correct prediction by chance alone). These metrics were computed from the “confusionMatrix” function in the caret R package version 6.0-92 [43].

## 3. Results

### 3.1. Overview of Weather Conditions during the Study

**Figure 1:**
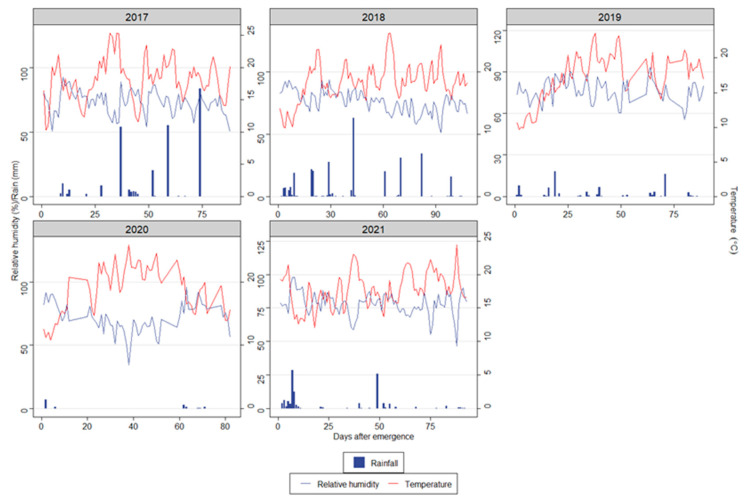
The weather conditions during the cropping seasons (2017–2021). The variables of daily mean relative humidity (RH, %) and accumulated rain (mm) are represented in Y1, and daily mean temperature (%) is shown in Y2. Days after emergence were the number of days after 50% of emergence. The emergence dates were 16 May (2017), 1 June (2018), 4 June (2019), and 10 June (2020 and 2021) (Table S1).

The daily weather conditions (daily mean temperature, daily mean relative humidity, and daily accumulated rainfall) during growing seasons are shown in Figure 1. The hotter and drier year was 2020. On the other hand, the coldest crop seasons were 2019 and 2021. A higher amount of rainfall was recorded in 2017 and 2018.

### 3.2. Daily Alternaria Conidia Concentration and Crop Phenology

**Figure 2:**
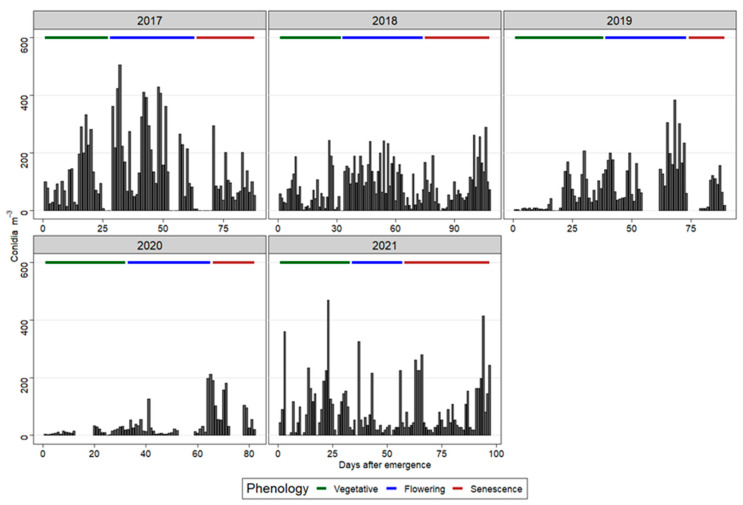
Daily conidia concentrations and phenological stages (vegetative, flowering, and senescence) in different cropping seasons. The emergence dates were 16 May 2017, 1 June 2018, 4 June 2019, and 10 June (2020 and 2021) (Table S1).

Daily conidia concentrations in the air at different phenological stages during the study years are shown in Figure 2. The conidia concentration varied between the five crop seasons. The 2020 season recorded the lowest conidia concentration (2476 conidia/m^3^) compared to the other years. The 2018 season had the highest value of conidia concentration (9264 conidia/m^3^) (Table S2). Finally, the 2017 season had the second highest conidia concentration (7873 conidia/m^3^).

According to the phenological stage, the highest number of conidia were found during the flowering (2017–2019) and senescence (2020) stages. In 2021, most conidia were trapped in the vegetative stage (Figure 2; Table S2). The conidia trapped during these stages accounted for >45% (2017–2019), 52% (2020), and 54% (2021) of the total (Table S2).

### 3.3. Correlation Structure via Graphical Model of the Hourly Data Set

**Figure 3:**
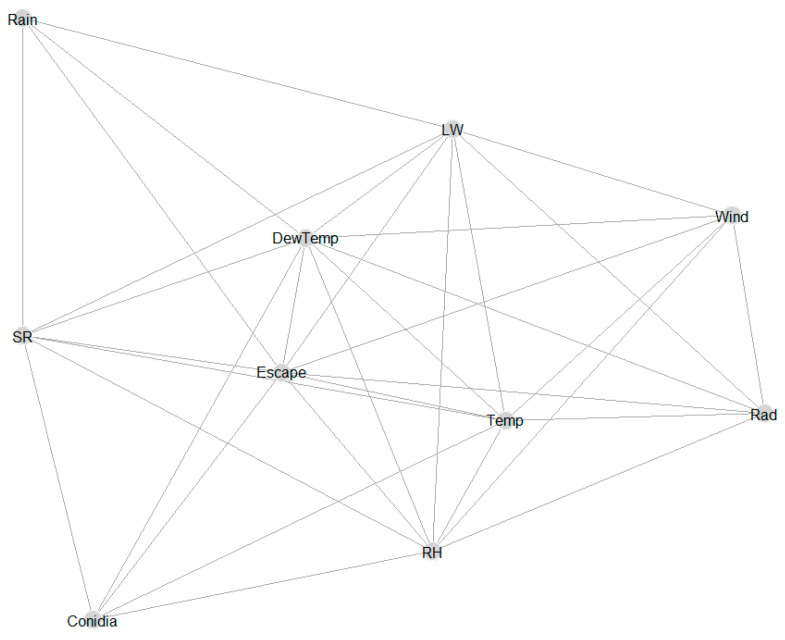
A graphical model showing the conditional dependence of different hourly weather variables and hourly concentrations of *A. solani* and *A. alternata* represented by the conidia variable. Variables that are connected by a line are conditionally dependent or correlated significantly and vice versa. The weather variables were relative humidity (RH), temperature (Temp), wind speed (Wind), dew temperature (DewTemp), leaf wetness (LW), solar radiation (Rad), and rainfall (Rain). The probability that conidia will be released is identified by the variable spore release (SR) and the probability that conidia will escape from the canopy is named escape (Escape).

The results showed significant correlations between some of the hourly weather variables (Figure 3). Five weather parameters (SR, DewTemp, Escape, Temp, RH) were directly connected to hourly conidia (conidia variable), showing the relevance of these weather parameters in explaining hourly patterns of conidia.

**Figure 4:**
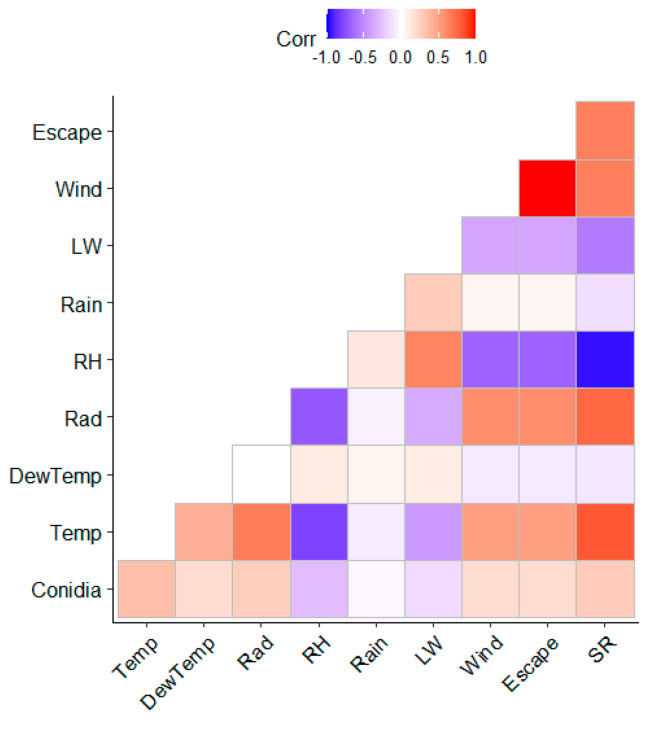
Heatmap with Spearman correlations between hourly conidia concentration and weather parameters. The selected weather variables were relative humidity (RH), temperature (Temp), wind speed (Wind), dew temperature (DewTemp), leaf wetness (LW), solar radiation (Rad), and rainfall (Rain).

The Spearman correlations between hourly conidia and weather variables are shown in Figure 4. Conidia were positively correlated with Temp, Rad, Escape, Wind, and SR. On the contrary, RH and LW showed a negative relationship with the conidia.

### 3.4. The Influence of the Weather Conditions per Hour on Alternaria Conidia

**Figure 5:**
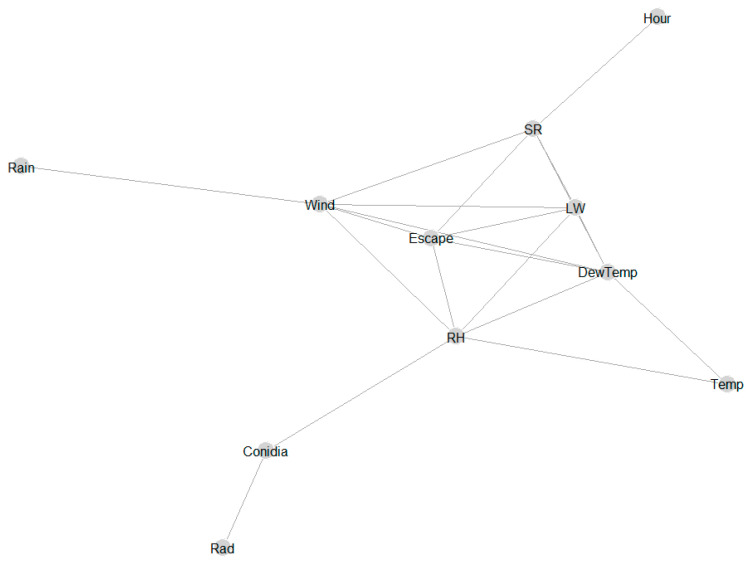
A graphical model showing the conditional dependence of weather variables and conidia concentrations at each hour in the day. Variables that are connected by a line are conditionally dependent or correlated significantly and vice versa. The weather variables were relative humidity (RH), temperature (Temp), wind speed (Wind), dew point temperature (DewTemp), leaf wetness (LW), solar radiation (Rad), and rainfall (Rain). The probability that conidia will be released is identified by the variable spore release (SR), and the probability that conidia will escape from the canopy is named escape (Escape).

Figure 5 summarizes the dependence among weather variables and conidia in each hour. Only SR was directly connected to the variable representing the hour of the day. The conidia concentration in a given hour was directly influenced by the RH and Rad of the hour, but not by the hour of the day variable (hour).

**Figure 6:**
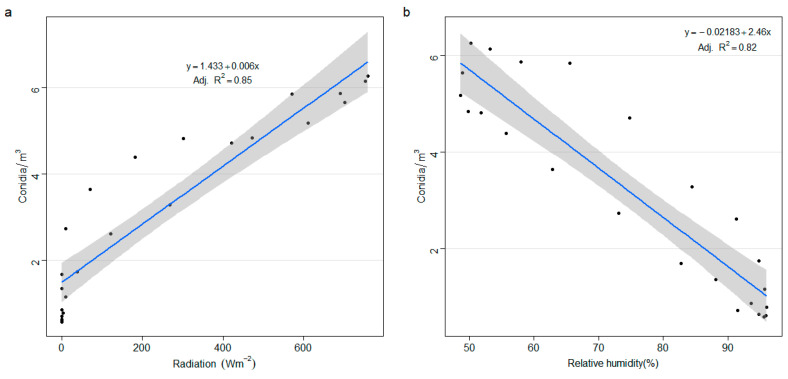
Linear regression graphs with confidence bounds of solar radiation (Radiation) (**a**) or Relative humidity (**b**) and conidia. The grey area surrounding the regression line represents the 95% confidence interval.

As shown in Figure 6, linear regression using Rad and RH as independent variables explained over 80% of the variation in conidia concentration per hour. Here, Rad was positively related to conidia concentration, while RH had a negative relation. Moreover, a fitted model including both Rad and RH as independent variables showed a significant effect on both weather variables, as well as their interactions on conidia concentration per hour (Table 1). The model explained 98% of the variation in conidia concentration per hour.

**Figure 7:**
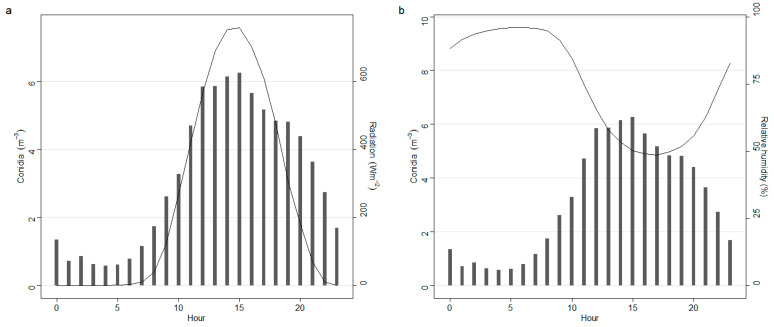
Intra-diurnal variations among *Alternaria* conidia and the most influenced weather parameters, namely solar radiation (Radiation) (**a**) and relative humidity (**b**). Each bar represents each hour of 24 h in a day.

Figure 7 summarizes the intra-diurnal pattern of conidia concentration together with the most relevant weather parameters according to the graphical model in Figure 5. Generally, conidia concentration began to rise from 8:00 am, and this coincided with a rise in Rad (>200 W/m^2^). This increase in conidia also coincided with a drop in RH (<90%) (Figure 6). The hours (i.e., 12:00–16:00) with the highest Rad and lowest RH were associated with the highest conidia concentration.

### 3.5. Analysis of Daily Data and Spearman Correlation between Alternaria Conidia and Weather Variables

**Figure 8:**
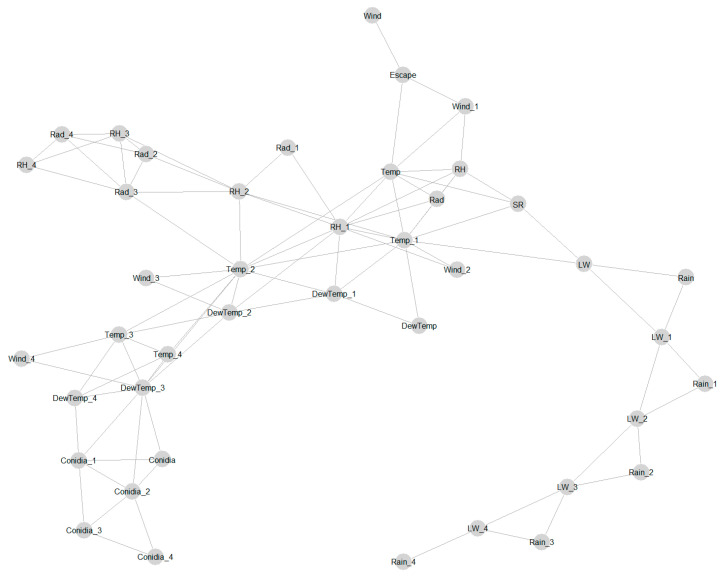
Graphical model showing conditional dependence/relationships between different daily weather variables and conidia on different days. The weather variables were temperature (Temp), dew temperature (DewTemp), relative humidity (RH), wind speed (Wind), spore escape (Escape), spore release (SR), leaf wetness (LW), solar radiation (SR), and rainfall (Rain). The variable (weather and conidia) numbers 1, 2, 3, and 4 indicate measurements taken 1, 2, 3, and 4 days ago, whereas those without numbers were measured or recorded on the current day.

The graphical model with the daily data set also showed a strong conditional dependence between the weather variables (Figure 8). Moreover, the graphical model showed a strong interdependence between the variables representing conidia (Figure 8). The present day’s conidia were directly connected to the conidia of the previous two days. Moreover, the weather variable that was directly connected to conidia was DewTemp. The dew temperature four days ago (DewTemp_4) influenced on the dew temperature of the following day (DewTemp_3) and the conidia concentration of two days after (conidia_1). In addition, the DewTemp_3 variable was directly related to the conidia concentration of the three subsequent days (conidia_2; conidia_1; conidia). Furthermore, the daily concentration of conidia was influenced by the concentration of the previous days, as shown by the connections in Figure 8.

**Figure 9:**
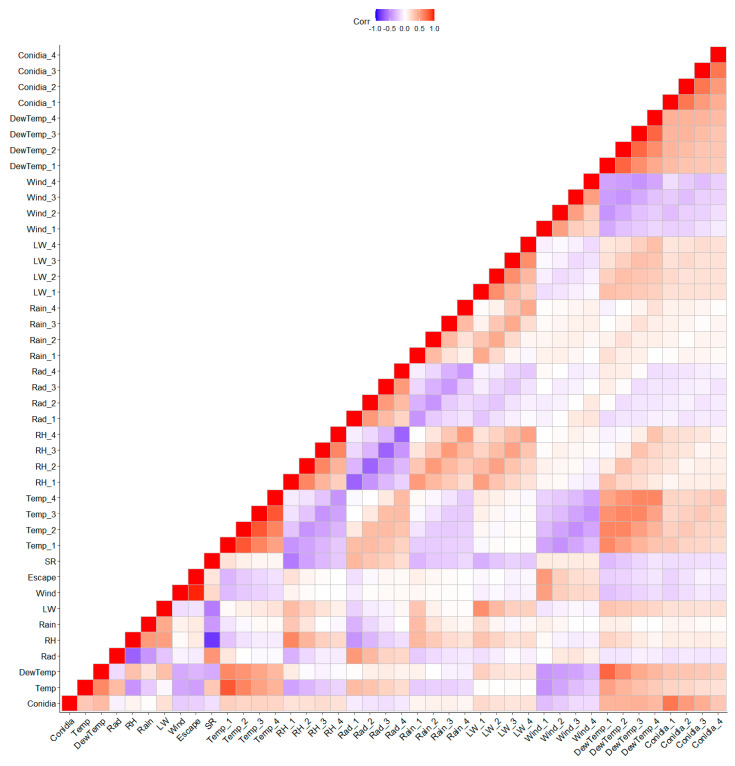
Heatmap with Spearman correlations between daily conidia concentration and weather parameters. The selected weather variables were relative humidity (RH), temperature (Temp), wind speed (Wind), dew temperature (DewTemp), leaf wetness (LW), solar radiation (Rad), and rainfall (Rain). The variable (weather and conidia) numbers 1, 2, 3, and 4 indicate measurements taken 1, 2, 3, and 4 days ago, whereas those without numbers were measured or recorded on the current day.

In general, conidia on the current day were more strongly correlated with conidia from the previous days (Figure 9). However, the strongest positive correlations were found between the conidia of the current and immediate past day. The conidia in the present day had the strongest positive correlation with DewTemp 1 and 2 days ago, compared to the other weather variables. In contrast, a stronger negative correlation was found between the past 1 day’s wind and conidia.

### 3.6. Application of Machine Learning Algorithms to Predict Daily Alternaria Conidia Levels and Optimization of Hyperparameters

The results of the cross-validation for optimizing the ML algorithms and graphical outputs of the models are shown in the Supplementary Materials (Figures S1–S10).

The optimal cp for the CART models were 0.03 (without conidia) and 0.48 (with conidia) (Figures S1 and S2). The tree-based model without winnowing was used for building the C5.0 model for the data without conidia. On the other hand, a rule-based model with winnowing was the best C5.0 model for the data with conidia. The KNN algorithm was built with a K value of 23 (without conidia) and 13 (with conidia). The optimal node size for the RF models were 1 (without conidia), and 8 (with conidia).

#### 3.6.1. Variable of Importance

The CART identified dew temperature (2 days ago) as the most important variable, as this variable represented the root of the tree in the absence of conidia in the data (Figure S3). In contrast, when the conidia were in the data set, the conidia on the immediate past day were the most important variable in the CART model (Figure S4). The RF model also ranked dew temperature four days ago (data without conidia) and the conidia on one day ago (data with conidia) as the most important variables.

#### 3.6.2. Evaluation of Model Performance

The accuracies, as well as the kappa statistics of the ML algorithms, are shown in Table 2. The RF model had the highest accuracy and kappa with the data without conidia, whereas the CART model was the most accurate when conidia were included in the ML process. In general, the models were more accurate (i.e., higher accuracy and kappa) when conidia from the previous days were included in the learning process. The only exception was the KNN algorithm, which had a lower accuracy and kappa in the presence of conidia compared to when conidia were excluded (Table 2).

Except for the KNN algorithm, there was a marked improvement in the kappa statistics when data from the previous days were included in the ML process. Model evaluation based on the kappa statistics showed that the models that did not include conidia were no better than a random guess, as their kappa statistics value was less than 0.5. In contrast, when the models included conidia from the previous days, the models were better than a random guess, as evidenced by their kappa statistics being greater than 0.5 (Table 2).

#### 3.6.3. Overview of the Wining Algorithm (CART)

The CART, which was the best model, predicted a meaningful conidia level when conidia on the previous day were at least 8.5 conidia/m^3^ (Figure S4).

## 4. Discussion

While early blight epidemics are usually initiated from overwintering inoculum in the soil [7], the subsequent disease development in the field is mainly caused by airborne conidia of *A. solani* and *A. alternata*. Therefore, understanding the factors that influence the airborne conidia of these pathogens is important. Accordingly, the goal of this study was to understand the key factors that influence the airborne conidia of the *A. solani* and *A. alternata* on the ambient growth of a potato crop, as well as to predict via ML the risk of a high pressure of inoculum that causes new reinfections.

Most of the counted conidia were captured during the reproductive stage. Several studies reported that the reproductive stage, which starts with flowering, generally, marks the point when the potato crop becomes susceptible to early blight and, thus, this fact supports profuse sporulation [10,44,45,46]. Abuley and Nielsen [1], in their maturity-based model, showed that fungicide application should only start during the reproductive stage, as this is the stage the crop is susceptible to early blight. In contrast to our result, Van der Waals et al. [6] found abundant conidia during crop senescence or harvest. This discrepancy with our study might be because we considered *A. solani* and *A. alternata*, whereas Van der Waals et al. [6] considered only *A. solani*. However, *A. alternata* is more abundant in the air, and its inclusion in our study might have influenced the conidia concentration.

Our study showed that the hour of the day matters less to the dispersal of conidia. Rather, the weather conditions (i.e.**,** solar radiation and RH) that characterize an hour are the major influence on conidia dispersal per hour. Indeed, the linear regression with RH and solar radiation explained 98% of the variation in intra-day conidia concentration. Practically, our results suggest a better prediction of the conidia in a given hour via the use of the weather variables that characterize the hour, but not the hour itself. Similar relationships among conidia and weather parameters (e.g.**,** as low RH and high solar radiation) were found by other authors [6,10,14,47]. Indeed, on rainless days, it is common to experience higher solar radiation and lower RH during the afternoon period of the day. It must also be noted that the reported low conidia dispersal during the night might be due to the weather conditions (low solar radiation and high RH) during these night times. Bardei et al. [48] also suggested that night times record fewer conidia compared to the daytime because of the unfavorable weather conditions for conidia release and dispersal (i.e.**,** low temperature and wind speed, and high RH) at night.

The unique approach adopted by this study of considering several variables, including the hour of the day in the analysis (such as in the graphical model) enabled us to arrive at this robust conclusion. Previous studies analyzed single weather variables and conidia per hour, and this might have masked their ability to arrive at the true determinant of the variation in conidia concentration in a day.

The fact that there was a strong correlation between conidia from previous days suggests the conidia trapped in a given day is unlikely to have been dispersed the same day. This also suggests that attempts to link conidia and the weather variables in a given day might result in a spurious correlation with no practical significance. For this reason, in this study, the weather conditions from the previous days in data analysis were included. As shown in the results, conidia on a given day was strongly correlated with previous weather variables. The weather variable with the strongest effect on conidia per day was dew temperature. Perhaps the fact that dew temperature is a good measure of dryness could explain its strong association with conidia. Indeed, high conidia concentrations are always associated with dryness. Nevertheless, it must be noted that this result is subject to the other variables used in the graphical model. The graphical model showed the conditional dependence or correlation, and these results might change if some variables are also changed. These results agree with Cowgill et al**.** [5] who proposed the use of dew (i.e.**,** dew severity value) for estimating early blight risk in the TOMCAST model. With this modification, the disease rate was reduced with fewer fungicide applications.

Whiles understanding the factors that influence the conidia concentration in the air is important, it is, perhaps, more relevant to use these factors for predicting the conidia concentrations in the air. Such predictions could play a vital role in integrating conidia concentration into the current disease forecasting models for early blight, which as yet do not include a sub-model for predicting airborne conidia. Our approach for predicting airborne conidia was based on classifying the total conidia per day as a meaningful level (M) or unmeaningful level (UM) when daily conidia levels were higher or lower than 10 conidia/m^3^, respectively. This level has previously been considered in other studies to forecast *Alternaria* concentrations [22]. Moreover, our field observations suggest that this threshold is the critical level of infection.

While ML algorithms are becoming increasing attractive for modelling big data, the correct choice of ML algorithm for the specific purpose is critical. This study evaluated a range of widely used ML algorithms for classification problems. The output of the ML shows that the inclusion of conidia from the previous days was critical for achieving a better prediction of conidia levels. As shown earlier in this study, the present day’s conidia were strongly linked to conidia from the previous day. Therefore, it is not surprising to arrive at a better prediction when conidia from the previous days were included in the ML process. To the best of our knowledge, our study is the first to achieve such a significantly high prediction accuracy of conidia level with ML.

The random forest (RF) algorithm has been touted as a better model for classification [21,29,30] because its ensembles several decision trees which, thus, improves its prediction strength. However, it was not the case in our study. When conidia were included in the ML process, the CART model emerged as the best model. It is, however, unclear to us what might have caused this higher prediction with the CART model compared to the RF model.

Although we had a small data set, our results have provided a strong basis for integrating aerial conidia into forecasting the risk of early blight. By simply classifying a day as risky (meaningful conidia level) or not (unmeaningful conidia level), models, such as TOMCAST, can be modified to improve risk assessment.

## 5. Conclusions

This study showed that conidia per hour is influenced by the weather conditions that characterize the hour, but not the hour of the day. Specifically, the RH and solar radiation were the most relevant weather parameters to explain the concentration of conidia per hour. Dew point temperature was the weather variable with the strongest effect on conidia per day. An improved prediction of conidia level was achieved via ML algorithms when conidia of previous days is considered in the analysis. Among ML algorithms, the CART model showed the best accuracy. Although more years of study are needed, these results can be useful to understand early blight epidemics on potato crop and increase the accuracy of developing forecast models for sustainable agriculture.

## Figures and Tables

**Table 1:** Coefficients of a combined linear regression analysis in which the weather variables that had the strongest effect on conidia were used to predict conidia concentration according to the hour in the day. Abbreviations are as follows: RH (relative humidity); Rad (solar radiation); RH: Rad (interactions between relative humidity and solar radiation).

	Estimate	Std. Error	t Value	*p*-Value
(Intercept)	7.815	0.517	15.123	<0.0001 **
RH	−0.073	0.006	−12.483	<0.0001 **
Rad	−0.006	0.001	−4.445	<0.0001 **
RH:Rad	0.0001	0.000	7.824	<0.0001 **

** *p* < 0.01.

**Table 2:** Metrics of different machine learning algorithms for predicting the conidia level per day with data sets that either included conidia or not.

	Algorithm ^a^	Kappa	Accuracy	CI ^b^
With conidia	C5.0	0.60	0.85	0.78–0.92
	CART	0.62	0.86	0.78–0.92
	KNN	0.40	0.79	0.700.86
	RF	0.51	0.83	0.76–0.91
Without conidia	C5.0	0.38	0.79	0.70–0.87
	CART	0.35	0.78	0.69–0.86
	KNN	0.41	0.80	0.71–0.88
	RF	0.43	0.80	0.74–0.89

^a ^The following machine algorithms were tested: classification regression trees (CART), C5.0 decision tree (C5.0), k-nearest neighbour (KNN), and random forest (RF). ^b^ The 95% confidence interval of the accuracy of the models.

## Data Availability

Not applicable.

## Supplementary Materials

The following supporting information can be downloaded at: https://www.mdpi.com/article/10.3390/s22187063/s1, Figure S1. Cross-validation to test a range of complexity parameters for the classification and regression tree (CART) model without *Alternaria* conidia; Figure S2. Cross-validation to test a range of complexity parameters for the classification and regression tree (CART) model with *Alternaria* conidia; Figure S3. A decision tree based on the classification and regression tree (CART) model with the data set without *Alternaria* conidia. UM and M represents meaningful (<10 conidia/m^3^) and meaningful (≥10 conidia/m^3^). The weather variable Wind_1, DewTemp_2, and Rad_4, represent wind (on 1 previous day), dew temperature (2 previous days), and solar radiation (4 previous days), respectively; Figure S4. A decision tree based on the classification and regression tree (CART) model with the data set with *Alternaria* conidia. UM and M represent unmeaningful (<10 conidia/m^3^) and meaningful (≥10 conidia/m^3^). The weather variable conidia_1 represent conidia in immediate past day; Figure S5. Cross-validation for the selection of the best model for the C5.0 decision tree model with the data set without *Alternaria* conidia; Figure S6. Cross-validation for the selection of the best model for the C5.0 decision tree model with the data set with *Alternaria* conidia; Figure S7. The variable of importance from the random forest model with the data set that did not include *Alternaria* conidia; Figure S8. The variable of importance from the random forest model with the data set that included *Alternaria* conidia; Figure S9. Cross-validation to select the optimal number of neighbors in the k-nearest neighbor (KNN) algorithm. The data used here did not include *Alternaria* conidia; Figure S10. Cross-validation to select the optimal number of neighbors in the k-nearest neighbor (KNN) algorithm. The data used here included *Alternaria* conidia; Table S1. Dates of the main phenological phases by growing season. DAE: days after emergence; Table S2. Total *Alternaria* conidia by growing season and distribution of conidia in percentage during the main phenological phases.

## References

[1] Abuley I.K., Nielsen B.J. (2017). Evaluation of models to control potato early blight (*Alternaria solani*) in Denmark. Crop Prot..

[2] Meno L., Escuredo O., Rodríguez-Flores M.S., Seijo M.C. (2021). Looking for a sustainable potato crop. Field assessment of early blight management. Agric. For. Meteorol..

[3] Abuley I.K., Nielsen B.J. (2019). Integrating cultivar resistance into the TOMCAST model to control early blight of potato, caused by *Alternaria solani*. Crop Prot..

[4] Meno L., Escuredo O., Rodríguez-Flores M.S., Seijo M.C. (2020). Modification of the tomcast model with aerobiological data for management of potato early blight. Agronomy.

[5] Cowgill W.P., Maletta M.H., Manning T., Tietjen W.H., Johnston S.A., Nitzsche P.J. (2005). Early blight forecasting systems: Evaluation, modification, and validation for use in fresh-market tomato production in northern New Jersey. HortScience.

[6] Van Der Waals J.E., Korsten L., Aveling T.A.S., Denner F.D.N. (2003). Influence of environmental factors on field concentrations of *Alternaria solani* conidia above a South African potato crop. Phytoparasitica.

[7] Abuley I.K., Nielsen B.J., Hansen H.H. (2019). The influence of crop rotation on the onset of early blight (*Alternaria solani*). J. Phytopathol..

[8] Aira M.J., Rodríguez-Rajo F.J., Jato V. (2008). 47 Annual records of allergenic fungi spore: Predictive models from the NW Iberian Peninsula. Ann. Agric. Environ. Med..

[9] Maya-Manzano J., Muñoz-Triviño M., Fernández-Rodríguez S., Silva-Palacios I., Gonzalo-Garijo A., Tormo-Molina R. (2016). Airborne *Alternaria* conidia in Mediterranean rural environments in SW of Iberian Peninsula and weather parameters that influence their seasonality in relation to climate change. Aerobiologia.

[10] Escuredo O., Seijo-Rodríguez A., Meno L., Rodríguez-Flores M.S., Seijo M.C. (2019). Seasonal dynamics of *Alternaria* during the potato growing cycle and the influence of weather on the early blight disease in north-west Spain. Am. J. Potato Res..

[11] Rodríguez-Rajo F.J., Iglesias I., Jato V. (2005). Variation assessment of airborne *Alternaria* and *Cladosporium* spores at different bioclimatical conditions. Mycol. Res..

[12] Konopińska A. (2004). Monitoring of *Alternaria* Ness and *Cladosporium* Link airborne spores in Lublin (Poland) in 2002. Ann. Agric. Environ. Med..

[13] Peternel R., Culig J., Hrga I. (2004). Atmospheric concentrations of *Cladosporium* spp. and *Alternaria* spp. spores in Zagreb (Croatia) and effects of some meteorological factors. Ann. Agric. Environ. Med..

[14] Grinn-Gofroń A., Bosiacka B., Bednarz A., Wolski T. (2018). A comparative study of hourly and daily relationships between selected meteorological parameters and airborne fungal spore composition. Aerobiologia.

[15] Skelsey P., Kessel G., Holtslag A., Moene A., Van Der Werf W. (2009). Regional spore dispersal as a factor in disease risk warnings for potato late blight: A proof of concept. Agric. For. Meteorol..

[16] Meredith D. (1963). Violent spore release in some fungi imperfecti. Ann. Bot..

[17] Escuredo O., Seijo M.C., Fernández-González M., Iglesias I. (2011). Effects of meteorological factors on the levels of *Alternaria* spores on a potato crop. Int. J. Biometeorol..

[18] Munuera Giner M., Carrión García J., Navarro Camacho C. (2001). Airborne *Alternaria* spores in SE Spain (1993-98). Grana.

[19] Fernández-González M., Ribeiro H., Pereira J., Rodríguez-Rajo F., Abreu I. (2019). Assessment of the potential real pollen related allergenic load on the atmosphere of Porto city. Sci. Total Environ..

[20] Grinn-Gofroń A., Strzelczak A., Wolski T. (2011). The relationships between air pollutants, meteorological parameters and concentration of airborne fungal spores. Environ. Pollut..

[21] Grinn-Gofroń A., Nowosad J., Bosiacka B., Camacho I., Pashley C., Belmonte J., De Linares C., Ianovici N., Manzano J.M.M., Sadyś M. (2019). Airborne *Alternaria* and *Cladosporium* fungal spores in Europe: Forecasting possibilities and relationships with meteorological parameters. Sci. Total Environ..

[22] Vélez-pereira A.M., Linares C.D. (2019). Logistic regression models for predicting daily airborne *Alternaria* and *Cladosporium* concentration levels in Catalonia (NE Spain). Int. J. Biometeorol..

[23] Cramer S., Kampouridis M., Freitas A.A., Alexandridis A.K. (2017). An extensive evaluation of seven machine learning methods for rainfall prediction in weather derivatives. Expert Syst. Appl..

[24] Rhee J., Im J. (2017). Meteorological drought forecasting for ungauged areas based on machine learning: Using long-range climate forecast and remote sensing data. Agric. For. Meteorol..

[25] Aybar-Ruiz A., Jiménez-Fernández S., Cornejo-Bueno L., Casanova-Mateo C., Sanz-Justo J., Salvador-González P., Salcedo-Sanz S. (2016). A novel Grouping Genetic Algorithm–Extreme Learning Machine approach for global solar radiation prediction from numerical weather models inputs. Sol. Energy.

[26] Fragni R., Trifirò A., Nucci A., Seno A., Allodi A., Di Rocco M. (2018). Italian tomato-based products authentication by multi-element approach: A mineral elements database to distinguish the domestic provenance. Food Control.

[27] Fang K., Shen C., Kifer D., Yang X. (2017). Prolongation of SMAP to Spatiotemporally Seamless Coverage of Continental U.S. Using a Deep Learning Neural Network. Geophys. Res. Lett..

[28] Bochenek B., Ustrnul Z. (2022). Machine Learning in Weather Prediction and Climate Analyses—Applications and Perspectives. Atmosphere.

[29] Wen L., Bowen C., Hartman G. (2017). Prediction of short-distance aerial movement of *Phakopsora pachyrhizi* urediniospores using machine learning. Phytopathology.

[30] Deng Y., Cheng X., Tang F., Zhou Y. (2022). The control of moldy risk during rice storage based on multivariate linear regression analysis and random forest algorithm. JUSTC.

[31] Wiesner-Hanks T., Wu H., Stewart E., DeChant C., Kaczmar N., Lipson H., Gore M.A., Nelson R.J. (2019). Millimeter-Level Plant Disease Detection From Aerial Photographs via Deep Learning and Crowdsourced Data. Front. Plant Sci..

[32] Liu J., Wang X. (2021). Plant diseases and pests detection based on deep learning: A review. Plant Methods.

[33] Galán S.C., González P.C., Teno P.A., Vilches E.D. (2007). Manual de Calidad y Gestión de la Red Española de Aerobiología.

[34] Hack H., Gall H., Klamke T., Meier U., Stauss R., Witzenberg A. (1993). Phänologische entwicklungsstadien der Kartoffel (*Solanum tuberosum* L.). Nachr. Dtsch. Pflanzenschutzd..

[35] R Core Team (2022). R: A Language and Environment for Statistical Computing.

[36] Abuley I., Hansen J.G. (2022). Characterisation of the level and type of resistance of potato varieties to late blight (*Phytophthora infestans*). Phytopathology.

[37] Abuley I.K., Nielsen B.J., Labouriau R. (2018). Resistance status of cultivated potatoes to early blight (*Alternaria solani*) in Denmark. Plant Pathol..

[38] Abreu G.C.G., Labouriau R., Edwards D. (2010). High-Dimensional Graphical Model Search with the gRapHD R Package. J. Stat. Softw..

[39] Kassambara A. (2019). ggcorrplot: Visualization of a Correlation Matrix Using ‘ggplot2’; R Package Version 0.1.3. https://CRAN.R-project.org/package=ggcorrplot.

[40] Nwanganga F., Chapple M., Nwanganga F., Chapple M. (2020). Decision Trees. Practical Machine Learning in R.

[41] Nwanganga F., Chapple M., Nwanganga F., Chapple M. (2020). k-Nearest Neighbors. Practical Machine Learning in R.

[42] Nwanganga F., Chapple M., Nwanganga F., Chapple M. (2020). Improving Performance. Practical Machine Learning in R.

[43] Kuhn M. (2022). Caret: Classification and Regression Training.

[44] Shtienberg D. *Alternaria* diseases of potatoes: Epidemiology and management under Israeli conditions. Proceedings of the Euroblight Workshop.

[45] Abuley I., Nielsen B.J., Bødker L., Nielsen G.C. Timing the application of fungicides to control potato early blight (*Alternaria solani*) in multi-location field trials in Denmark. Proceedings of the Euroblight Workshop.

[46] Meno L., Abuley I.K., Escuredo O., Seijo M.C. (2022). Suitability of Early Blight Forecasting Systems for Detecting First Symptoms in Potato Crops of NW Spain. Agronomy.

[47] Iglesias I., Rodríguez-Rajo F.J., Méndez J. (2007). Evaluation of the different *Alternaria* prediction models on a potato crop in A Limia (NW of Spain). Aerobiologia.

[48] Bardei F., Bouziane H., Trigo M.d.M., Ajouray N., El Haskouri F., Kadiri M. (2017). Atmospheric concentrations and intradiurnal pattern of *Alternaria* and *Cladosporium* conidia in Tétouan (NW of Morocco). Aerobiologia.

