# Supplementary material for: Importance of Meteorological Parameters and Airborne Conidia to Predict Risk of Alternaria on a Potato Crop Ambient Using Machine Learning Algorithms
Source: Sensors (Basel). 2022 Sep 18;22(18):7063. doi: 10.3390/s22187063 (PMC9500921; doi:10.3390/s22187063)
Supplement: Supplementary file 1 [file sensors-22-07063-s001.zip › sensors-1899685-supplementary.pdf]

Supplementary information

Table S1. Dates of the main phenological phases by growing season. DAE: days after emergence.

	Date (DAE)				
	**2017**	**2018**	**2019**	**2020**	**2021**
**Planting**	22 Apr	15 May	16 May	26 May	22 May
**Emerging**	16 May (1)	1 Jun (1)	4 Jun (1)	10 Jun (1)	10 Jun (1)
**Flowering**	13 Jun (29)	4 Jul (34)	13 Jul (40)	13 Jul (34)	14 Jul (35)
**Start of senescence**	19 Jul (65)	15 Aug (76)	17 Aug (75)	14 Aug (66)	7 Aug (59)
**End of senescence**	12 Aug (89)	14 Sept (106)	1 Sept (90)	31 Aug (83)	26 Aug (78)

Table S2. Total *Alternaria* conidia by growing season and distribution of conidia in percentage during the main phenological phases.

	**2017**	**2018**	**2019**	**2020**	**2021**
**Total *Alternaria* conidia**	7873	9264	6399	2476	6444
***Alternaria* conidia (%)**					
**Vegetative stage**	37	22	21	16	54
**Flowering stage**	45	49	68	32	25
**Senescence stage**	18	29	11	52	21

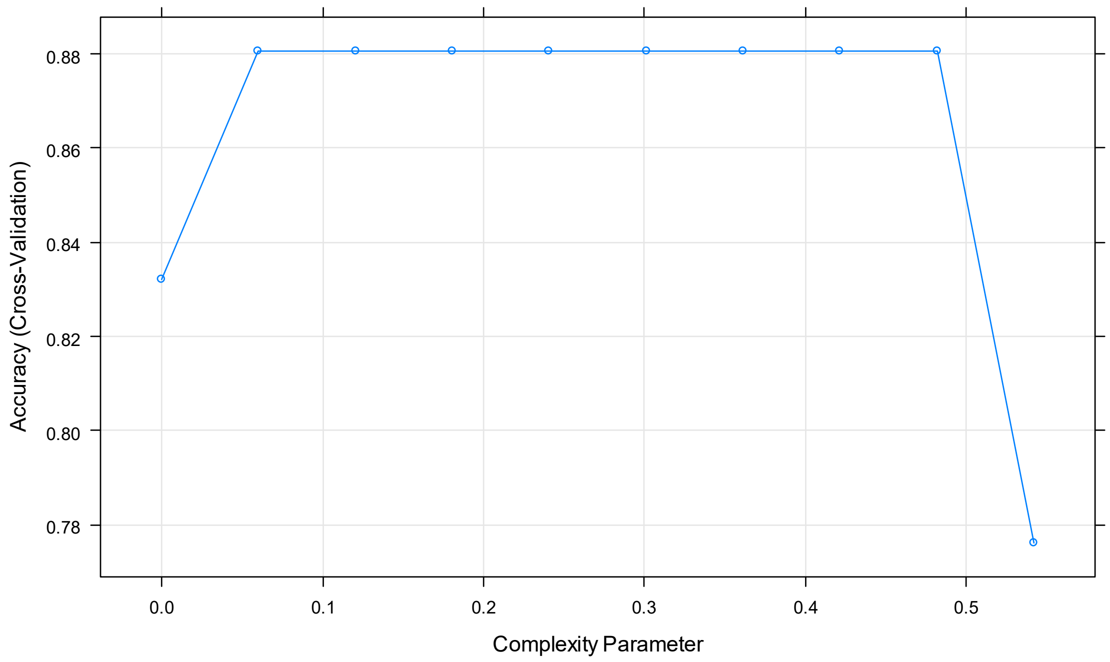

Figure S1. Cross-validation to test a range of complexity parameters for the classification and regression tree (CART) model without *Alternaria* conidia.

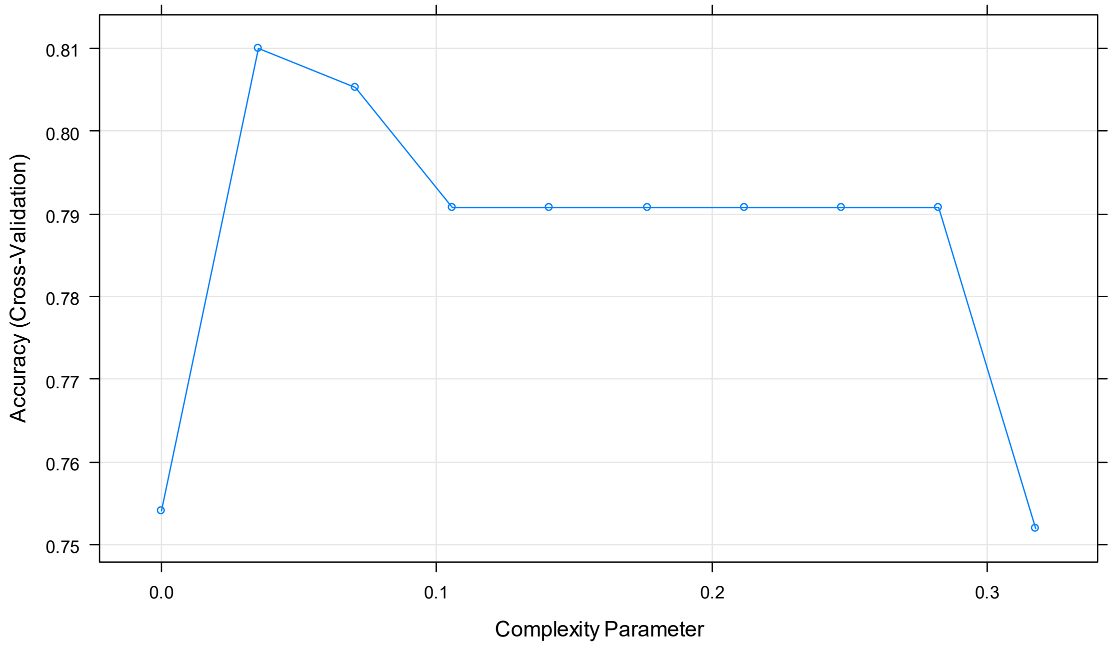

Figure S2. Cross-validation to test a range of complexity parameters for the classification and regression tree (CART) model with *Alternaria* conidia.

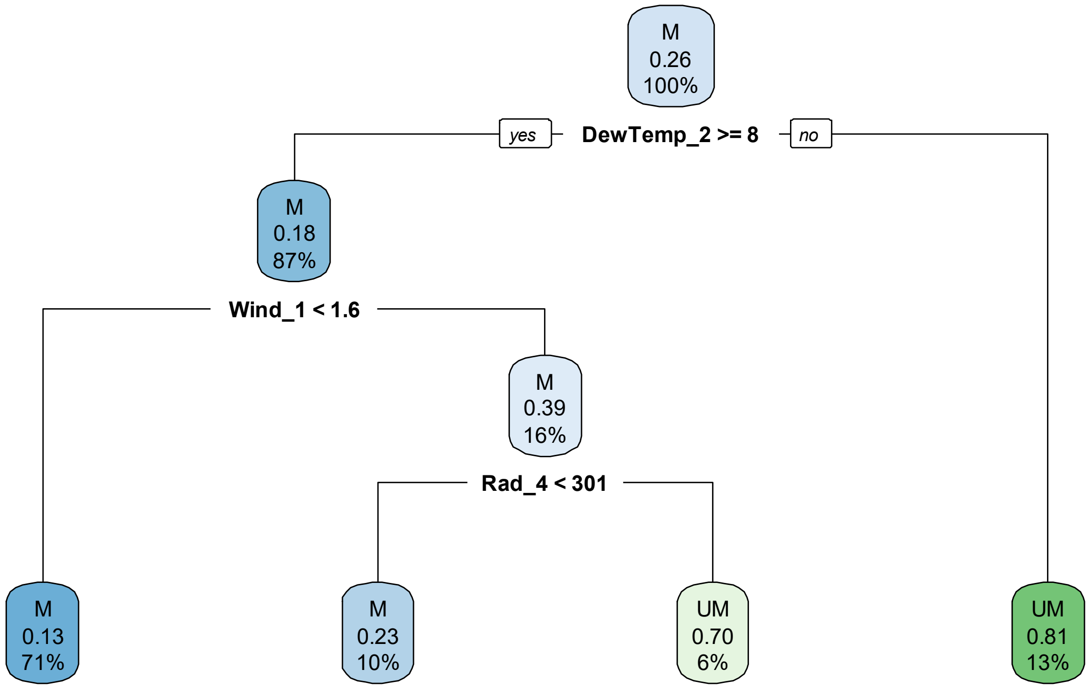

Figure S3. A decision tree based on the classification and regression tree (CART) model with the data set without *Alternaria* conidia. UM and M represents meaningful (<10 conidia/m^3) and meaningful (≥ 10 conidia/m^3). The weather variables Wind_1, DewTemp_2, and Rad_4, represent wind (on 1 previous day), dew temperature (2 previous days), and solar radiation (4 previous days), respectively.

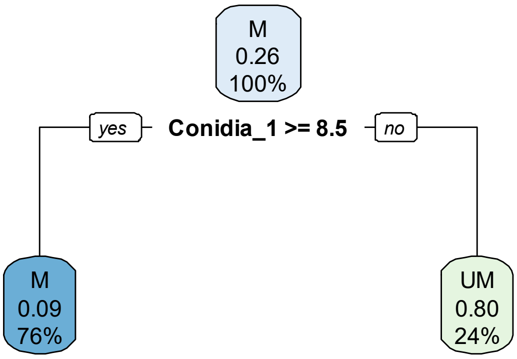

Figure S4. A decision tree based on the classification and regression tree (CART) model with the data set with *Alternaria* conidia. UM and M represent meaningful (<10 conidia/m^3) and meaningful (≥ 10 conidia/m^3). The weather variable conidia_1, represent conidia in the immediate past day.

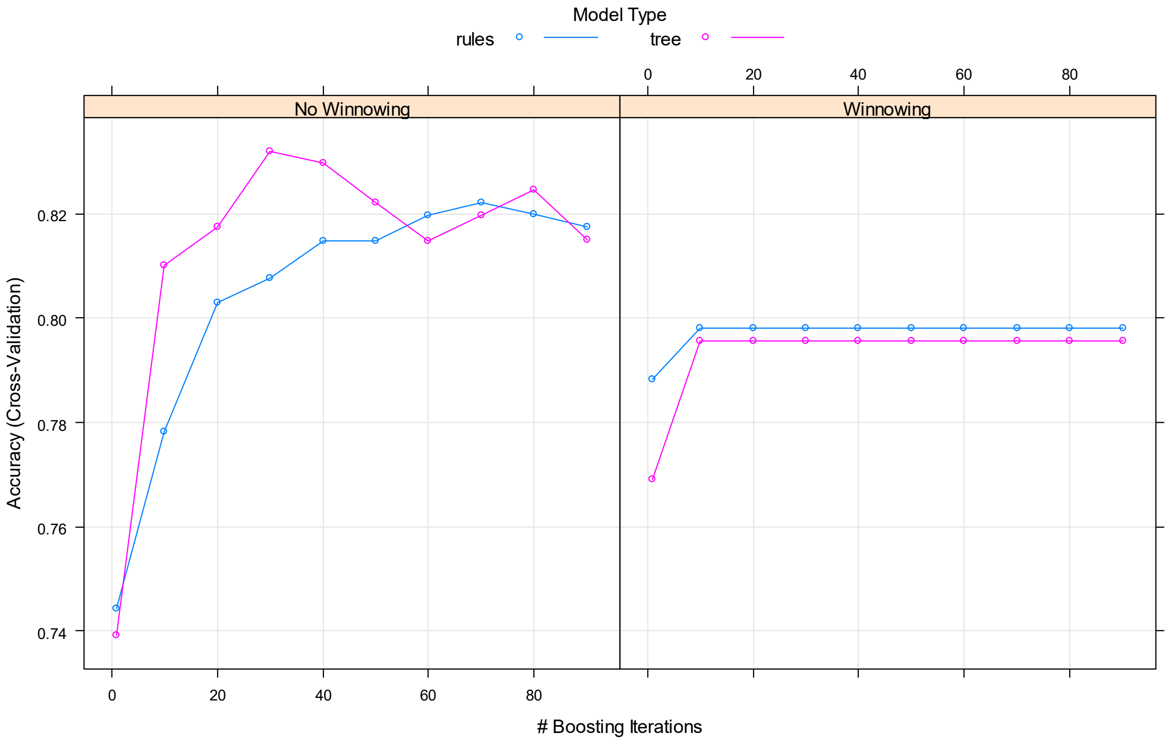

Figure S5. Cross-validation for the selection of the best model for the C5.0 decision tree model with the data set without *Alternaria* conidia.

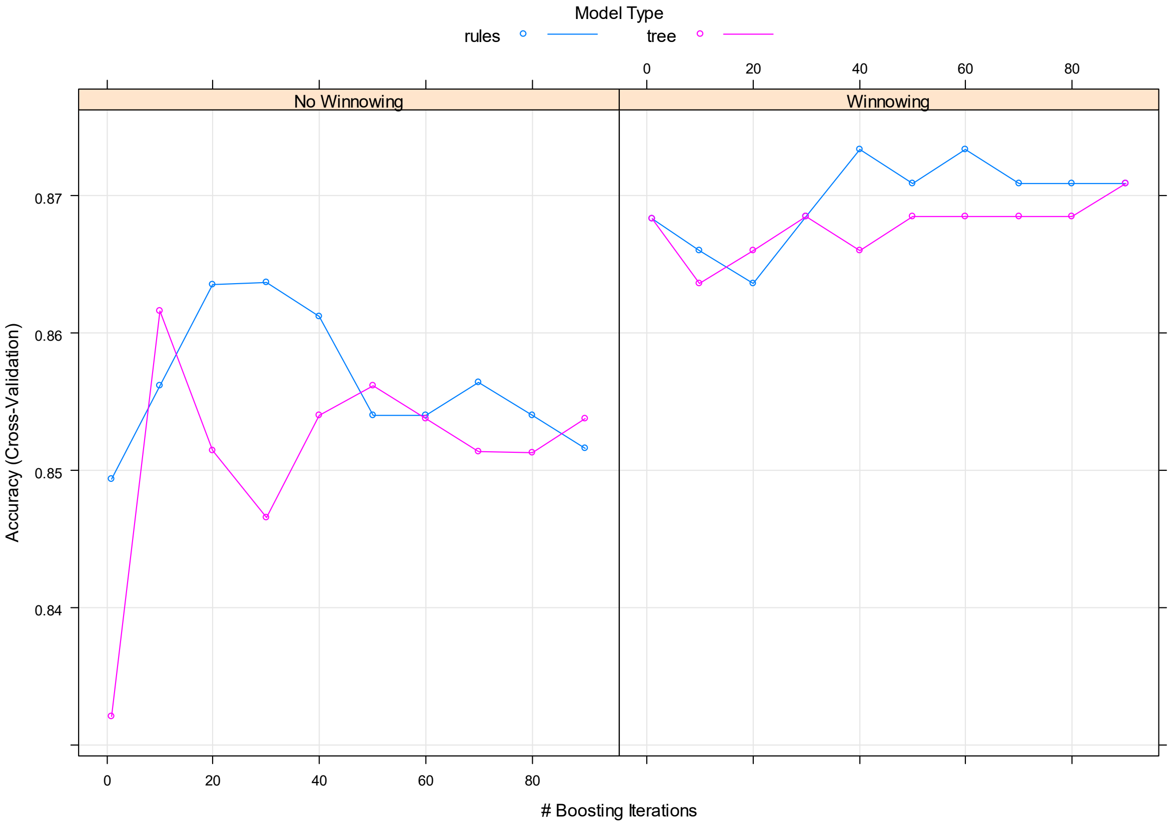

Figure S6. Cross-validation for the selection of the best model for the C5.0 decision tree model with the data set with *Alternaria* conidia.

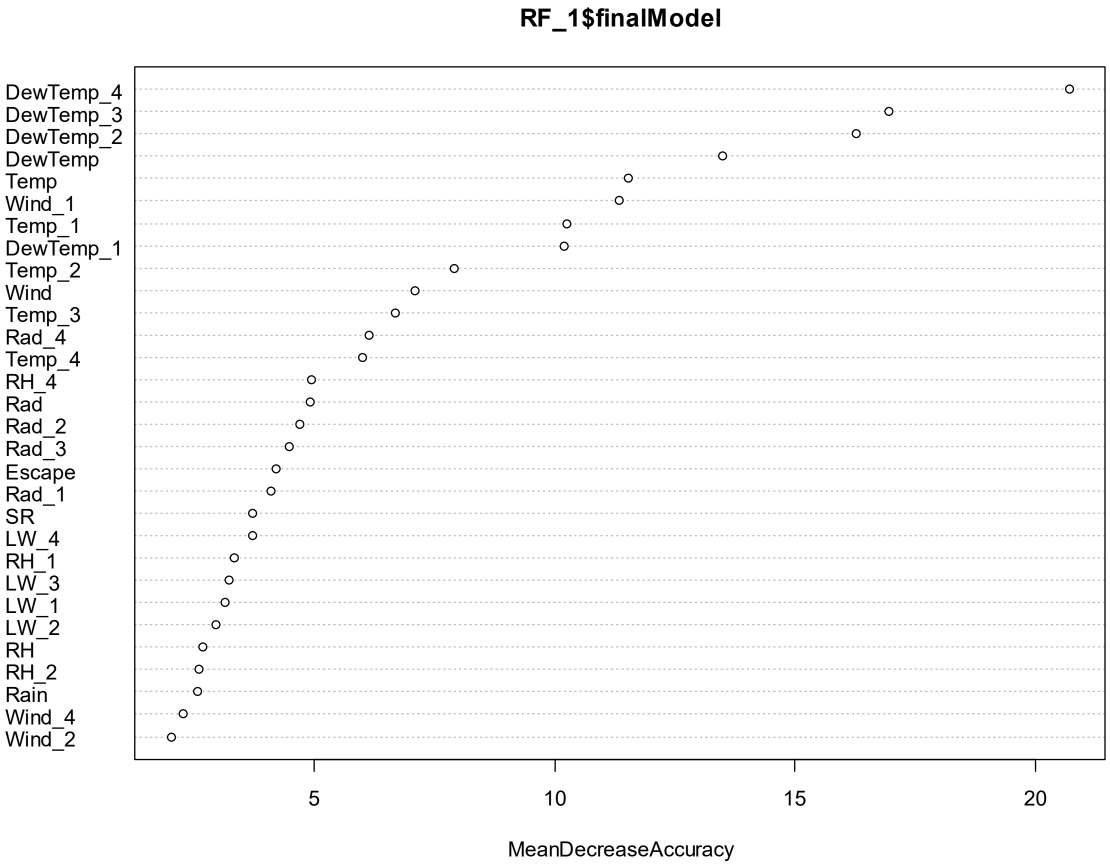

Figure S7. The variable of importance from the random forest model with the data set that did not include *Alternaria* conidia.

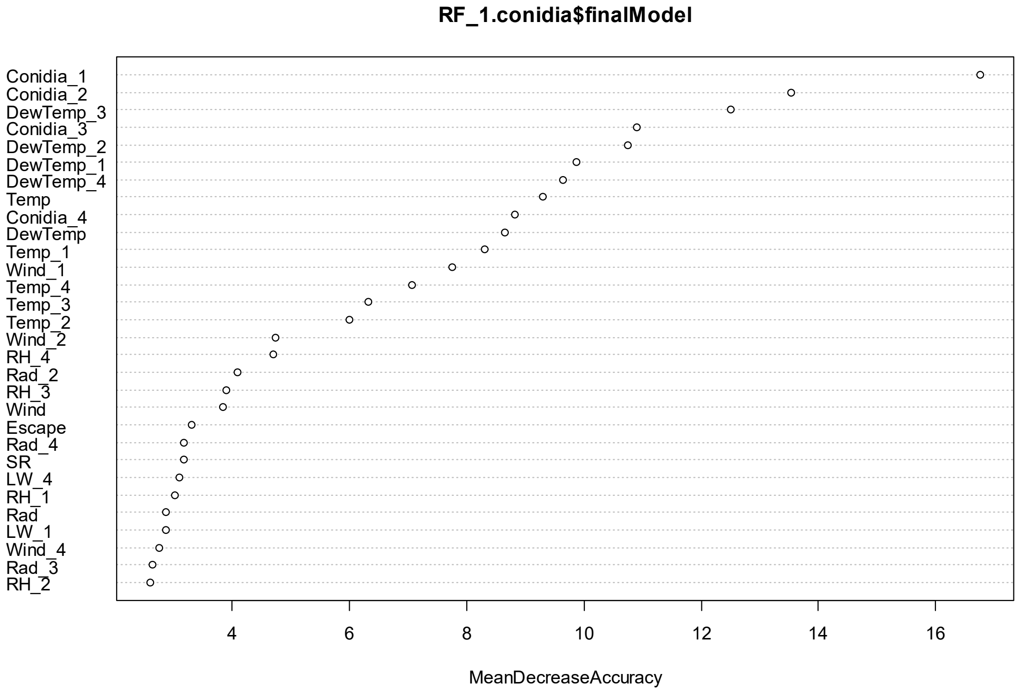

Figure S8. The variable of importance from the random forest model with the data set that included *Alternaria* conidia.

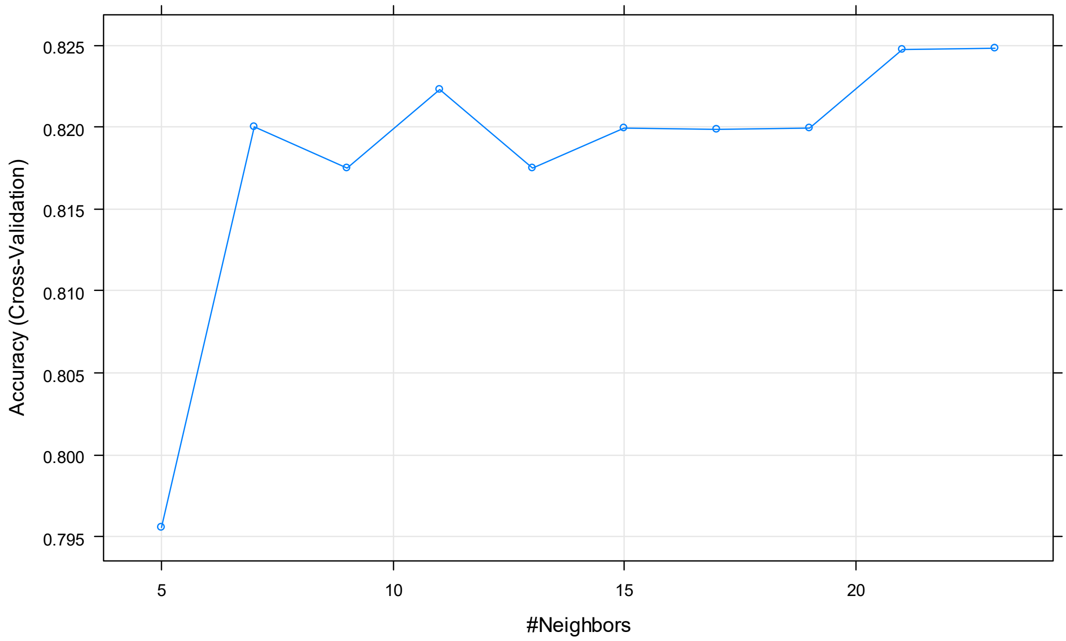

Figure S9. Cross-validation to select the optimal number of neighbors in the K-nearest neighbor (KNN) algorithm. The data used here did not include *Alternaria* conidia.

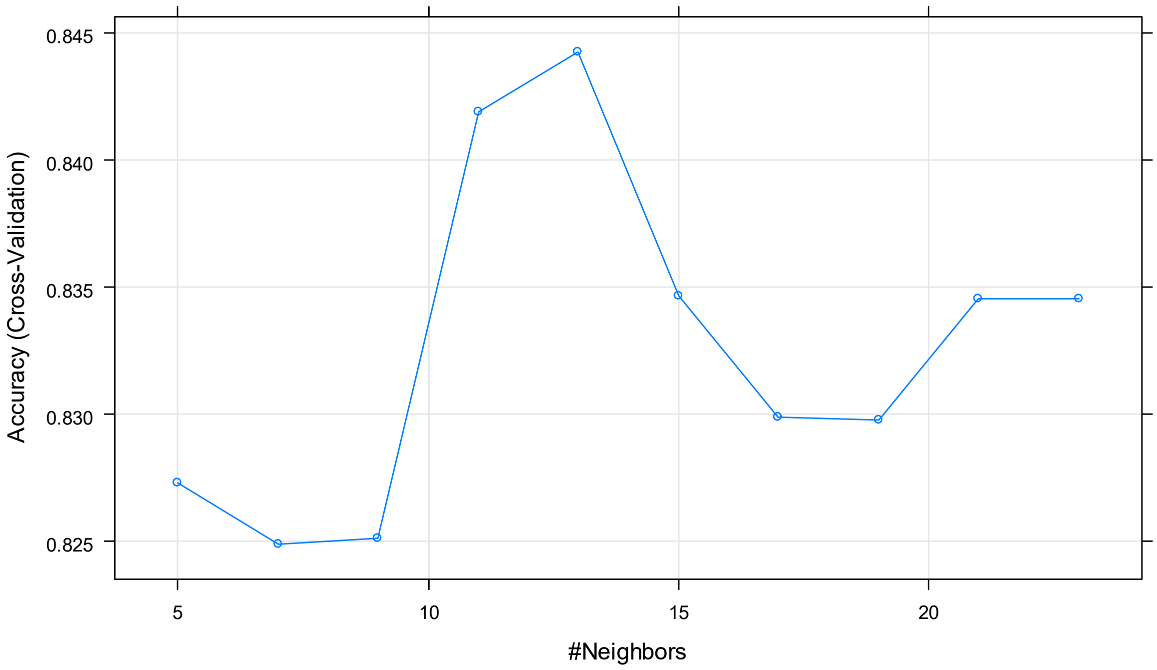

Figure S10. Cross-validation to select the optimal number of neighbors in the K-nearest neighbor (KNN) algorithm. The data used here included *Alternaria* conidia.
